# Integrative Base Ontology for the Research Analysis of Alzheimer’s Disease-Related Mild Cognitive Impairment

**DOI:** 10.3389/fninf.2021.561691

**Published:** 2021-02-04

**Authors:** Alba Gomez-Valades, Rafael Martinez-Tomas, Mariano Rincon

**Affiliations:** Department of Artificial Intelligence, Universidad Nacional de Educación a Distancia (UNED) Madrid, Spain

**Keywords:** ontology, MCI, Alzheimer’s disease, neuropsychological tests, neurodegenerative disease, ontology design

## Abstract

Early detection of mild cognitive impairment (MCI) has become a priority in Alzheimer’s disease (AD) research, as it is a transitional phase between normal aging and dementia. However, information on MCI and AD is scattered across different formats and standards generated by different technologies, making it difficult to work with them manually. Ontologies have emerged as a solution to this problem due to their capacity for homogenization and consensus in the representation and reuse of data. In this context, an ontology that integrates the four main domains of neurodegenerative diseases, diagnostic tests, cognitive functions, and brain areas will be of great use in research. Here, we introduce the first approach to this ontology, the Neurocognitive Integrated Ontology (NIO), which integrates the knowledge regarding neuropsychological tests (NT), AD, cognitive functions, and brain areas. This ontology enables interoperability and facilitates access to data by integrating dispersed knowledge across different disciplines, rendering it useful for other research groups. To ensure the stability and reusability of NIO, the ontology was developed following the ontology-building life cycle, integrating and expanding terms from four different reference ontologies. The usefulness of this ontology was validated through use-case scenarios.

## Introduction

In the last few decades, there has been a true revolution in the volume and complexity of the data created in the life sciences and, with them, in the possibilities of studying such data (Hoehndorf et al., 2015). These data are scattered, under different formats, and generated by different technologies, so they are very heterogeneous and widely dispersed (Decety and Cacioppo, 2010; Costa, 2014). Thus, the creation of an adequate infrastructure that allows the standardization, exchange, and sharing of information as key objectives for the success of research efforts (Burgun and Bodenreider, 2008), which, in turn, is essential to improve the quality of life of patients (Zhang et al., 2014).

In this context, the development of ontologies has been established as one of the most appropriate solutions in the biomedical domain (Blake and Bult, 2006), especially in neurology, where mental processes are described at multiple levels of abstraction (Hastings et al., 2012). In particular, mild cognitive impairment (MCI) has attracted special attention in research because it is a transitional phase between normal aging and dementia, and can be an early indicator of Alzheimer’s disease (AD) and other neurodegenerative disorders (El-Gamal et al., 2018; Tavares-Júnior et al., 2019; Wasserman et al., 2020). However, the presence of MCI is not always clear, nor easy to identify; hence, methods capable of detecting it efficiently in its early stages are needed, and the development and improvement of data interoperability methods are recognized as essential for it (Zhang et al., 2014).

Various tests have been developed for the early diagnosis of MCI. These tests evaluate the state of patients in different ways: based on biological markers, brain imaging, or psychological and neuropsychological tests (NT). The former two options are expensive, invasive, and rely on highly specialized equipment, features that make them unsuitable as screening methods (Clark et al., 2014a). Psychological testing and NT lack these problems while maintaining comparable reliability to biomarkers testing (Klages et al., 2005; García-Herranz et al., 2020). Psychological testing focuses on detecting depressions, anxiety, apathy, and other behavioral and psychological symptoms of dementia (Scassellati et al., 2020), while neuropsychological testing is capable of measuring cognitive domains, such as memory, executive function, or attention (Hastings et al., 2014), and detecting their alterations. Both psychological and neuropsychological testing should be considered when assessing a subject’s cognitive status because behavioral changes can influence NT scores (García-Herranz et al., 2020; Wasserman et al., 2020). Therefore, they should be considered as cognitive assessment tools that allow the initial screening of patients based on their cognitive abilities and have the advantages of being non-invasive, versatile, and low cost. Besides, they are also suitable for monitoring the progression of the MCI (Tavares-Júnior et al., 2019).

In this particular study, we consider four interrelated domains, namely, diagnostic testing (particularizing in the case of neuropsychological testing to narrow the scope and make it more manageable), cognitive processes, brain areas, and neurodegenerative diseases, which also include the medical history of the subject. The underlying theory that relates them is that: (1) physical damage in different areas of the brain is correlated with different alterations in the cognitive functions of patients; (2) these alterations are detected by NT, as they are reflected as different types of failures (signs) when performing each test; and (3) failures are related to different neuropsychological and neurodegenerative diseases. However, despite the great interrelationship between these domains, they are usually studied in a rather isolated way, prioritizing in each study certain areas against others.

Given that by focusing on a single domain or some fragments of them, part of the information involved in the characterization of neurodegenerative diseases is ignored, an ontology that integrates the knowledge of these four domains would be of great help to researchers and physicians involved in the investigation and early detection of MCI and neurodegenerative diseases in general. As we will see in the next section, various ontologies have been developed with that goal in mind; however, they are oriented to specific parts of the problem and, mainly, they are difficult to adapt to new projects, either because they have been designed for a specific project, or because of their overcomplexity for the problem in question. Hence the need for a new ontology with the necessary flexibility to be adapted to new projects, and that integrates the required knowledge with the least possible overlap.

In this context, we propose the Neurocognitive Integrated Ontology (NIO), as the first step toward this ontology. However, due to the broad scope of neurodegenerative diseases, in NIO we will focus on the modeling of MCI related to AD through NT, and their relations with cognitive functions and brain areas. This new ontology, which should be easily expandable and adaptable, will be developed from terms and properties represented in already existing ontologies, expanding it afterward with new terms and relationships to complete it. Thanks to these features, although NIO is framed in a broader project, to provide a conceptual model for the early detection of MCI with a high probability of conversion to AD, it can also serve as a basis that facilitates the integration of new terms from different domains, allowing the extension, reuse, and specialization of the ontology by other research groups that adapt it to their projects. This is reflected in the use cases described in the last part of the article, where we show how to adapt the ontology to easily model a database obtained from a longitudinal study for the early detection of MCI (García-Herranz et al., 2016; Díaz-Mardomingo et al., 2017), or how to extend NIO with another ontology that covers the imaging tests (IT) domain.

The article is structured as follows: in “Ontologies for Neurodegenerative Disease Research” section, we analyze the state-of-the-art ontologies developed for the study and early detection of neurodegenerative diseases. In “Ontology for the Analysis and Deep Interpretation of NT” section, we describe our proposal. In “Evaluation” section, two practical use cases are shown in which new knowledge is modeled and the ontology is expanded. Finally, in “Conclusion” section, we expose the conclusions and problems found in the development of the ontology.

## Ontologies for Neurodegenerative Disease Research

An ontology is a formal definition of classes, properties, and relationships between them that is framed within an area of knowledge. This allows homogenization and consensus in the representation of a domain (Trokanas and Cecelja, 2016), which facilitates the exchange of information by favoring the integration and recovery of heterogeneous data from different sources, and this, in turn, can improve the diagnosis and treatment of a disease (Mead, 2006).

However, the use of ontologies also has problems. The main and most immediate one is the low utilization of predefined terms, which causes redundancy and inconsistency problems, such as conflicts in the name of the terms, unstable references, and redundancy in the class hierarchy (Klein, 2001). To avoid these problems, a priority during the development of new ontologies should be to reuse already existing ontologies as much as possible, only adding new classes and instances when those concepts are not covered by any of the selected ontologies (Gómez-Pérez et al., 2004).

In this context of reuse, we analyze cutting-edge ontologies related to MCI with a high probability of evolving into AD and neuropsychological testing to select the ones closest to our goals. Table 1 shows an overview of the most relevant ontologies organized by the coverage of tasks they provide. Some ontologies were publicly available, others were not. In the latter case, they were evaluated from the article that described them.

**Table 1:** Summary of ontologies related to the understanding of Alzheimer’s disease (AD) and other neurodegenerative diseases.

Ontology name	Publicly available	Last update	Task coverage	Domain coverage	Upper ontology	Ontology reuse	Internal structure	Class hierarchy	Metadata
SWAN (Gao et al., 2006)	No	2009	General	Global	None	—	—	—	—
ND (Jensen et al., 2013)	Yes	2012	General	Global	BFO	High	Medium	High	High
NPT (Cox et al., 2013)	Yes	2013	Test modeling	Local (NT)	BFO	High	Medium	High	High
NDDO (Kostovska et al., 2019)	Yes	2019	General	Global	OBO	High	High	High	Medium
ADO (Malhotra et al., 2014)	Yes	2013	General	Global	BFO	Low	Medium	Medium	Medium
OntoNeuroLOG (Batrancourt et al., 2015)	Yes	2013	Test modeling	Local (D)	DOLCE	High	High	High	Low
MIND (Sanchez et al., 2011)	No	2011	Diagnosis	Local (D)	None	Low	—	—	—
Ontology-driven decision support system (Zhang et al., 2014)	No	2013	Diagnosis	Local (IT)	SNOMED CT	No	—	—	—
Multiagent (Ivascu et al., 2015)	No	2015	Diagnosis	Local (D)	None	No	High	Low	Low
AlzFuzzyOnto (Zekri et al., 2015)	No	2015	Diagnosis	Local (D)	None	Low	—	—	—

*It shows whether the ontology is publicly available, the date of their last update or article publication when the ontology is not publicly available, task and domain coverage, upper ontology used, degree of ontology reuse, the internal structure of the ontology, class hierarchy, and quantity and quality of metadata. Those aspects that could not be evaluated are marked with “–”*.

Ontologies were classified into three main categories according to the task coverage provided to the different domains involved in our task:

• General coverage: these ontologies provide a general representation of AD. They focus on the subject’s medical history, symptoms, diagnostic method, and treatment. However, as the scope is so broad, they do not delve into all subdomains.
• Test modeling: they focus on representing the knowledge of diagnostic tests. This knowledge ranges from resources and socio-demographic data to different tests, their results, and their meanings.
• Diagnosis: normally, these ontologies are created from scratch and specialize in a particular test or set of tests, making them difficult to reuse for other purposes. They usually constitute a subsystem within a larger one dedicated to the early diagnosis of MCI.

Other aspects of the ontologies that we evaluated, and the options we distinguished, were the following:

• Domain coverage: either they represented all aspects of a domain (global) or focused on a part (local) to help with some specific problems, such as diagnosis (D), IT, or NT.
• Upper or foundation ontologies: whether the ontology was built on some foundation ontology, i.e., Basic Formal Ontology (BFO; Arp and Smith, 2011), Open Biomedical Ontologies Foundry (OBO; Smith et al., 2007), DOLCE Foundation Ontology (DOLCE; Gangemi et al., 2002), SNOMED CT (Spackman et al., 1997), or no standard was followed.
• Degree of reuse of other ontologies: if the ontology is based on other ontologies as much as possible (High), is based on other ontologies in the more general classes (Medium), or only general guidelines are employed or there is no reutilization at all (Low).
• Internal Structure: ontologies with a high number of relationships and axioms (High), the average number of relationships and generally concentrated in more generic classes (Medium), or a low number of relationships and axioms in general (Low).
• Class Hierarchy: ontologies with usually more than 1,000 classes and deep nesting (High), less nesting depth (Medium), or less than 100 classes, and low nesting depth (Low).
• Metadata: ontologies with complete and detailed annotations (High), short, missing, or incomplete annotations (Medium), or almost total absence of annotations (Low).

### Ontologies for the General Coverage of Neurodegenerative Diseases

The Semantic Web Applications in Neuromedicine (SWAN) project of Gao et al. (2006) led to one of the first ontologies focused on the storage and contextualization of existing information on AD. According to the authors, SWAN provided a common standard, which allowed its use for physicians and researchers. The project was developed as an infrastructure that effectively integrated existing scientific knowledge about AD, allowing the construction of a semantic network of hypotheses, publications, and digital repositories at that moment (Ciccarese et al., 2008). SWAN was considered the reference repository for AD knowledge available on the web, but nowadays, this ontology and the associated application have been removed from all the repositories where they were stored.

The Neurological Disease Ontology (ND; Jensen et al., 2013) seeks to provide a framework to represent the most relevant aspects of neurodegenerative diseases that can help in their study and treatment. ND provides a set of controlled classes that describe factors, such as range, signs and symptoms of neurodegenerative diseases, and evaluations, diagnoses, and medical interventions that have been found in the course of clinical practice. ND also allows linking and extending it to other existing ontologies of the same domain. However, as the ontology tries to cover such a broad domain, it is very general for our purposes. Also, it has an over-complexity problem, which stems from the fact that ND has inherited classes that belong to heterogeneous domains with little or no relationship to the target domain.

The Alzheimer Disease Ontology (ADO; Malhotra et al., 2014) attempts to provide the widest possible coverage of the different aspects of the AD domain in a structured way. This is one of the ontologies that cover most aspects, including diagnosis, treatment, and molecular mechanisms. Although it only has shallow coverage in some subdomains, ADO stands out in its coverage of cognitive processes. Like SWAN, ADO was designed to allow extraction and inference of the stored data through queries. However, the axiomatic system is difficult to interpret.

Finally, the Neurodegenerative Disease Data Ontology (NDDO; Kostovska et al., 2019) is an ontology that seeks a representation of data related to neurodegenerative diseases, focusing on AD and Parkinson’s disease. Its objective is to facilitate the semantic annotation of data related to diagnosis and disease progression to allow reasoners to infer new knowledge based on facts. NDDO presents a high degree of term reuse, which facilitates interoperability and reusability by other research groups.

### Ontologies for NT Representation

After the development of the ND ontology, the Neuro-Psychological Testing Ontology (NPT; Cox et al., 2013) was presented to extend and complement the ND ontology in the part of NT. It was also developed with the idea of facilitating its further expansion with new tests. This makes the NPT a very specialized and comprehensive ontology in that domain, which provides a large set of classes to represent and annotate a wide variety of NT and associated data, and also evaluate various domains of the cognitive function. However, NPT has the same problem of excessive complexity as ND, making it difficult to locate relevant classes.

Finally, OntoNeuroLOG (Batrancourt et al., 2015) focuses on the instruments used to evaluate the brain and its cognitive functions, as with tests, such as the Mini-Mental State Exam. It has been developed within the NeuroLOG project (Michel et al., 2010) to share evaluation results based on the instruments. Therefore, OntoNeuroLOG is a multilayer ontology organized in sub-ontologies or modules arranged in three levels of abstraction (abstract level of classes provided by DOLCE, generic and key concepts for each domain of interest provided by “core” domain ontologies, and domain-specific concepts) and it has a great internal structure, with different types of relationships, restrictions, and axioms defined between classes. However, the ontology focuses on brain IT rather than NT.

### Ontology-Oriented Diagnosis Systems

MIND ontology (Sanchez et al., 2011) was proposed as part of an ontology-based management system, and it complemented a reasoning system for decision making to help physicians in the early detection of AD. This project merged ontologies and a semantic reasoner able to infer logical consequences from a given set of facts and axioms. Among other concepts, MIND describes different diagnostic tests (neuropsychological, neurological, radiological, metabolomics, and genetic tests).

In Zhang et al. (2014), an ontology-driven decision support system was proposed for the diagnosis of MCI that sought to avoid subjectivity. The ontology focused solely on MRI for the detection of cerebral cortex thickness, as it is reduced in patients with MCI (Whitwell et al., 2008), and it ignored other methods such as NT.

The work of Ivascu et al. (2015) depicted a multi-agent ontology, to facilitate remote monitoring of patients at risk of developing cognitive impairment. In this work, a combination of ontology and a multi-agent system was used, in which a group of programs specialized in a task and able to work together (multi-agent system) collected and processed the data before comparing it with a database (the ontology) to issue a diagnosis. The ontology was developed as a disease-symptom-sensor system, so the multi-agent ontology could provide real-time information to physicians about patients. Because the ontology was completely focused on practical use, their terms were oriented to be relevant to their system, making them difficult to reuse in other settings.

Finally, Zekri proposed AlzFuzzyOnto (Zekri et al., 2015), an ontology-based on MIND that enabled the semantic representation of medical data for the diagnosis and support of AD. The idea is that a significant number of concepts that introduce uncertainty and inaccuracy in the model can be adequately represented by fuzzy classes, and those concepts can be linked by fuzzy relationships. The system created an AD fuzzy ontology that can be useful for diagnosis in real-life situations.

Of the ontologies reviewed, none simultaneously covered all four domains of NT, brain areas, mental functions, and AD. Furthermore, we had problems when trying to adapt the found ontologies to our objectives. Therefore, we considered as the best option to build a new ontology integrating all modules of interest from previous ontologies. The new ontology should also be easily adaptable and extensible.

## Ontology for the Analysis and Deep Interpretation of Nt

In this section, we present the design of a new ontology reusing the modules of interest previously modeled in other ontologies. For this, a comparison between the ontologies found was made as a first step, to select those whose modules best covered the domains to be modeled. Based on Table 1, which summarizes the analysis carried out to select the most appropriate domain ontologies for the diagnosis of MCI due to AD through the analysis of NT, we made the following decisions.

First, ND and NPT were very similar, both focusing on AD and other neurodegenerative diseases and their early detection. However, ND had a wider scope and lower depth, being a very generic ontology for our purpose. Therefore we preselected NPT, which provided fairly complete coverage of NT while keeping all relevant classes of ND.

The Multi-agent ontology was created from scratch without reusing any existing ontologies, so the class distribution diverged substantially from most of the other selected ontologies, which used BFO as the upper ontology. OntoNeuroLOG was left aside because, although promising and very complete, it was more focused on IT rather than NT, and like the Multi-agent ontology, the distribution of classes diverged substantially, as it used DOLCE as the upper ontology instead of BFO. ADO was selected because the domain related to AD was comprehensively covered. Finally, NDDO was ruled out due to a strong focus on brain imaging, a high degree of overlap with ND and ADO, and lower specificity. Therefore, NPT and ADO were selected because the NT and AD domains were more comprehensively covered. Unfortunately, we could not get the rest of the ontologies.

However, none of the listed ontologies covered the domains of mental functions and brain areas in sufficient detail, so we searched for two reference ontologies to complete both domains. For the domain of cognitive functions, the Mental Functioning (MF) Ontology (Hastings et al., 2012) was selected. To model the physical structure of the brain, we analyzed two ontologies, Uber Anatomy Ontology (UBERON; Mungall et al., 2012) and Foundation Model of Anatomy (FMA; Rosse and Mejino, 2008), both reference ontologies in anatomy. We selected FMA because it focused on the anatomy of the human body.

Once, we selected the ontologies, we proceeded with the development of NIO. For this purpose, Protégé 5.2^1^ was used. We made an initial alignment between the selected ontologies to: (1) choose the most relevant groups of terms within each of the domains; and (2) remove the rest with the main objective of centering the ontology within our scope, avoiding overloading it with classes of little relevance. Then, we created the structure of the ontology by integrating the clean modules.

The ontology was carefully inspected a second time, searching for redundancies not detected before and inconsistencies arising from the presence of the same term in different ontologies. The redundancy problem is due to the lack of reuse between ontologies, which causes duplicate terms to appear once or several times, in near or far positions, and with the same label or synonym. Correcting it is essential to prevent an inconsistent ontology.

The redundancy issue was also behind the decision to include only two ontologies for the AD and NT domains, as they covered both domains most comprehensively. Nonetheless, finding and removing redundancies was one of the longest steps. Depending on the original ontology, certain terms were given preference over others: FMA ontology had priority in terms related to the brain structure, MF ontology in terms related to cognitive processes, and NPT ontology in terms related to neurodegenerative disease and NT.

At this moment, we did an initial evaluation of the stability and pitfalls of the constructed ontology. Ontology stability was checked using two different methods: the Protégé reasoner HermiT1.3.8 and the online program OOPS! (Ontology Pitfall Scanner!^2^), which helps to detect the most common pitfalls that appear when developing ontologies. We first used the reasoner to detect inconsistent relationships between terms, and we corrected these inconsistencies. Next, we used OOPS! to identify the ontology pitfalls. We fixed both critical and important pitfalls, as well as minor pitfalls related to deficiencies in the structure of the ontology. Following, the ontology was evaluated again using the reasoner to check if the pitfalls correction had generated any inconsistency. This cycle was repeated until the reasoner raised no inconsistencies, and the pitfalls scanner showed no critical or serious pitfalls.

Next, new relationship terms with the necessary object properties were created using the relations established in the literature (Knopman and Petersen, 2014; Luna-Lario et al., 2015; Trojano and Gainotti, 2016; Müller et al., 2017) as a guide. For example, the classes of “Geriatric Depression Scale” and “Yesavage Depression Scale” was linked to the “inclusion criterion” class using the new property of “is used in,” and the class “Mild Cognitive Impairment” was linked to some evaluation tests using the property “is evaluated by.” Finally, a new evaluation of the ontology stability and pitfalls was performed after the new terms and relationships were established.

**Figure 1:**
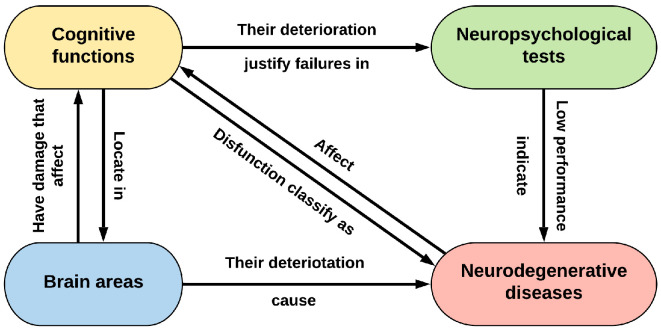
Scheme of the relationship between the four major domains of the ontology. It would also be completed with the other transversal information of interest, such as medical history, risk factors, or possible treatments.

In this way, we obtained a stable and structured ontology with the most relevant concepts of the four domains that could help to better understand the relationships within MCI (Figure 1) and deepen the interpretation of NT. The NIO ontology is available *via* BioPortal at https://bioportal.bioontology.org/ontologies/NIO.

Below is a summary of the actions carried out for the development of NIO:

• Search for ontologies.
• Analyze and compare the ontologies found.
• Select the most relevant ontologies for the project (ADO and NPT).
• Search for reference ontologies for domains not exhaustively covered by any of the previous ontologies [in our case, brain areas (FMA) and cognitive function (MF)].
• Remove non-relevant terms to avoid overloading the ontology.
• Integrate the ontologies and generate a new one.
• Remove redundant classes inside the resulting ontology.

The following criteria were used:

° In physical brain areas, the structure defined by the FMA will have priority over the other three, because it is the reference ontology of human anatomy.
° In cognitive functions, MF has priority over the others because it is the reference ontology in the domain of mental functions.
° Between ADO and NPT, the latter will have priority as it was developed with greater emphasis on standardization.

• Create new relationships between domains according to what has been found in the literature (neuropsychological testing, cognitive functions, brain areas, and AD).
• Evaluation of the ontology using a reasoner, pitfalls scanner, and through two use case evaluations.

## Evaluation

To illustrate the advantages of using NIO we show two practical examples focusing on two main problems we encountered when originally looking for suitable ontologies: the ability of the ontology to be able to model the knowledge from an external project, and the ability to the ontology of being able to be completed in its domains with modules from another ontology. In the first one, we have adapted NIO to a specific research study of early detection of MCI with a high probability of conversion to AD using NT. With this use case, we will show how the ontology can be easily adapted to a particular research project inside the AD domain and model its knowledge. In the second use case, we extend the ontology by incorporating new modules obtained from a different ontology, to show how the ontology can be extended both in existing domains and with new related domains.

### Use Case 1: Adapting the Ontology to a New Project

Given its ease of standardizing data and facilitating its use, linking ontologies to reasoners and machine learning systems are an increasingly common practice. However, there are still problems in adapting existing ontologies to research projects carried out by other research groups. This issue leads some researchers to develop their ontology capable of modeling the features of their project. That generates more heterogeneity, which is the opposite of what is intended with ontologies. In this use case, we show a practical example where we have adapted NIO to a specific research study, which requires expanding our ontology with new terms and properties to model and integrate their data. We used a longitudinal study carried out by the Faculty of Psychology of the National University of Distance Education [Universidad Nacional de Educación a Distancia (UNED)] focused on the combined use of different NT for the early detection of MCI with a high probability of conversion to AD (García-Herranz et al., 2016; Díaz-Mardomingo et al., 2017). This study seeks to discern which tests or test items are more descriptive in detecting MCI in early stages, before conversion to AD or other neurodegenerative diseases, and the possible influence of socio-demographic variables. This study is framed in the neurodegenerative testing domain. Therefore, NIO will be extended by adding the necessary relations and properties of this domain and instantiating the ontology with the information stored in the study database.

As a first step, the necessary categories for modeling the data within the ontology were identified in the database. Next, a list was created with the database terms and relationships to transfer to NIO. We tried to locate and use these classes and relations inside the NIO. When we did not find a term, we had to create it in the right place. For instance, we introduced tests that were not previously represented in NIO under the same category as the closest test. The new terms were the following: “Rey-Osterrieth Complex Figure Test” and “Barcelona Test” under “Cognitive Tests;” “Yesavage Depression Scale (Reduced version)” under “Geriatric Depression Scale;” “SESLAS” under “Mood Evaluation” and “Verbal fluency” and “TAVEC” under “Simple Word Test.” The other tests presented in the database, such as “Trail Making Tests A and B,” were already modeled in the ontology.

**Figure 2:**
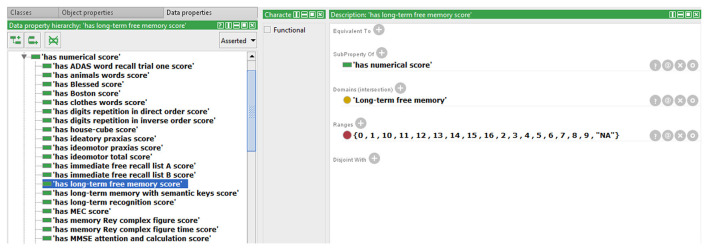
Example of defining a new data property element with its domain and range.

**Figure 3:**
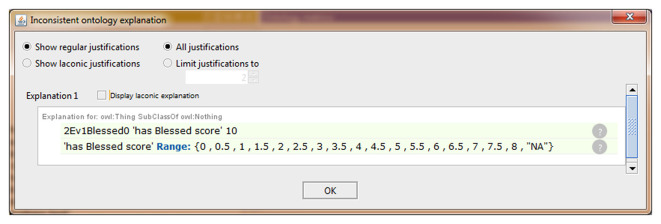
Example of an inconsistency in the instantiation detected by the HermiT reasoner.

**Figure 4:**
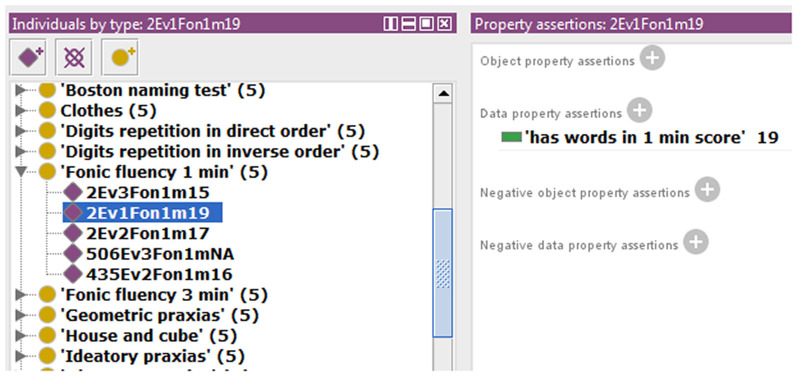
Example of instantiation in Neurocognitive Integrated Ontology (NIO), showing the value entered under instance 2Ev1Fon1m19, which corresponds to the score obtained by subject 2 in the first evaluation, in the 1-min phonetic fluency test.

We also extended the ontology to include some data property not previously modeled in NIO. An example that allows storing the scores obtained by each subject in the different test items is shown in Figure 2. All necessary Data Property was defined from scratch as the sub-property of “has a numerical score.” Because each test can only have one score for each subject and evaluation, a restriction to prevent more than one value per instance was implemented in the data property “has numerical score,” making it a functional property. All children of this data property inherited this characteristic. The classes that were related to the sub-properties of “has numerical score” were set in the “domain” section and the values allowed were set in “range.” In cases where the score was limited, as in the graphic tests, the exact values that could be achieved were specified, and where no range limits were defined, as in “fluency tests,” the range was defined as natural numbers. Also, an indicative value of data absence was added as a conjunction to discern cases with no score from those in which the score was not introduced in the ontology by mistake. Finally, the ontology was instantiated using some cases from the database to verify that this knowledge now can be modeled in NIO. It was also checked that restrictions worked correctly by adding some incorrect values or out of range values, which the reasoner marked as inconsistency, as can be seen in Figure 3. Figure 4 shows an example of a correct final instantiation in NIO, where the value introduced under the instance 2Ev1Fon1m19 corresponds to the score obtained in the 1-min phonetic fluency test by subject 2 in the first evaluation.

Defining properties so restrictively while modeling the relationships between the different terms, improved consistency by reducing the risk of certain errors going unnoticed when entering data, allowing data to be standardized by putting it in the same format.

### Use Case 2: Updating the Ontology Using a New Ontology

One of the key points in the development of ontologies is their ability to be extended and completed in their domains by integrating knowledge modeled in other ontologies. However, this is not always easy, usually due to the use of different standards and the lack of reusability between ontologies. This use case illustrates how to extend NIO classes in the IT field to incorporate that knowledge. IT is widely used in the early detection of MCI and the combination of IT and NT leads to more efficient diagnoses than using only one of those tests alone (Clark et al., 2014b). We chose the NDDO ontology for this use case, as it was one of the most recent ontologies in the field and had high reuse of previous ontologies.

As a preparatory step, we analyzed the NDDO ontology to identify those classes and modules that were inside the current scope of NIO and could therefore be used to extend the ontology. Both ontologies shared the same upper classes, which sped up the process of searching for suitable modules. After this initial analysis, a set of potential modules was selected. In the next step, we analyzed these modules in-depth to discard those that, although related to the global scope of our ontology, had a very high level of detail that did not correspond to the current state of development of NIO, i.e., all terms related to the diagnosis that was based on biomarkers tests.

As a final step before integrating the selected modules in NIO, they were checked to look for redundancies with classes already presented in NIO. When those redundancies appeared, the classes were compared and those which came from a more curated ontology or had more complete metadata were maintained.

After all revisions were completed, the extracted modules were integrated into NIO and a final evaluation was performed looking for redundancies that might not have been detected in the previous steps. Finally, the stability of the ontology was tested using the inner reasoner of Protégé. No inconsistencies arose.

**Figure 5:**
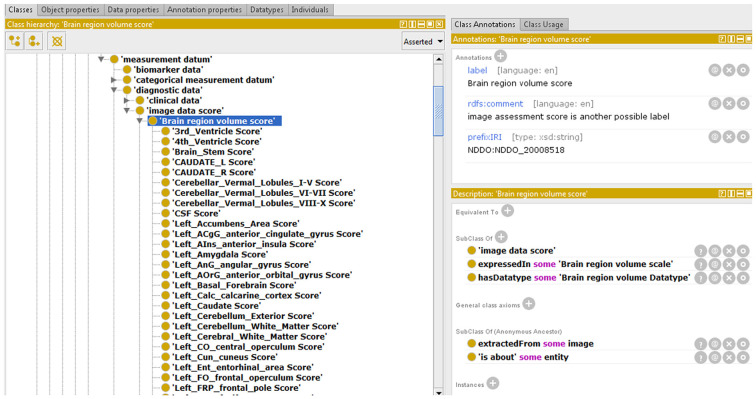
Partial view of an Neurodegenerative Disease Data Ontology (NDDO) module integrated with NIO.

This way, we integrated the NDDO modules corresponding to brain IT on NIO and, as an example, we show one of them, “Brain region volume score,” in Figure 5. We also used part of the NDDO modules to complete the NIO modules related to NT and neurodegenerative diseases in all those cases in which NDDO presented a more exhaustive coverage. We verified the structure and stability of NIO before uploading the new version into BioPortal.

## Conclusion

In this article, we have presented NIO, an ontology that constitutes the first step towards a unified ontology in which all knowledge present in the four domains of neuropsychological diseases, diagnostic tests, cognitive functions, and brain areas is modeled and integrated. This integration will help to gain a deeper understanding of neurodegenerative diseases and how they affect brain areas and cognitive functions. Also, as part of a machine learning-based system, this knowledge can be used to design more efficient (shorter) screening tests by analyzing the discriminating power (effectiveness in measuring the current state of different cognitive functions) of the different items that make up the tests.

To ensure the stability and reusability of NIO, the ontology was developed following the ontology-building life cycle, integrating and expanding terms coming from four different reference ontologies. During the development of NIO, several problems derived from the lack of reuse and consensus has been found. This led us to carefully check the ontology at two different stages, to identify and remove redundant terms and avoid inconsistencies.

We created NIO with the idea of overcoming the main problems we encountered in previous ontologies, specifically: (1) the focus on a specific part of the domain related to the early diagnosis of MCI; (2) the difficulty of adapting the ontology to an external project; and (3) the difficulty of expanding existing ontologies with new domains, or with new modules related to domains modeled. This way, NIO is an easily expandable ontology able to model new knowledge consistently, as has been demonstrated in the two use cases. In the first use case, we demonstrated how classes and properties corresponding to those NT used in the study that was not previously modeled in NIO could be easily added, checking afterward the relationships and restrictions modeled can detect data inconsistency. In the second use case, we showed how to integrate the knowledge available in another ontology to extend a domain in NIO. We expect to complete NIO with new classes and relationships that deepen the knowledge represented by: (1) linking neurodegenerative diseases with their corresponding relevant terms in the other domains; (2) adding new elements and alterations, such as macrography and micrography, which are detected in the tests but to date they have not been taken into account in the quantitative evaluation; and (3) relating these alterations to the corresponding cognitive functions and neurodegenerative diseases.

## Data Availability Statement

The datasets presented in this study can be found in online repositories. The names of the repository/repositories and accession number(s) can be found in https://bioportal.bioontology.org/ontologies/NIO.

## Author Contributions

AG-V contributed to the literature review and made the first version of the NIO. RM-T contributed to the revision and correction of the NIO. AG-V and RM-T wrote the manuscript. MR gave valuable advice and assisted in editing the manuscript. All authors contributed to the article and approved the submitted version.

## Conflict of Interest

The authors declare that the research was conducted in the absence of any commercial or financial relationships that could be construed as a potential conflict of interest.

## Footnotes

^1^http://protege.stanford.edu

^2^http://oops.linkeddata.es/
